# Translational Validation of Personalized Treatment Strategy Based on Genetic Characteristics of Glioblastoma

**DOI:** 10.1371/journal.pone.0103327

**Published:** 2014-08-01

**Authors:** Young Taek Oh, Hee Jin Cho, Jinkuk Kim, Ji-Hyun Lee, Kyoohyoung Rho, Yun-Jee Seo, Yeon-Sook Choi, Hye Jin Jung, Hyeon Suk Song, Doo-Sik Kong, Ho Jun Seol, Jung-Il Lee, Yeup Yoon, Sunghoon Kim, Do-Hyun Nam, Kyeung Min Joo

**Affiliations:** 1 Samsung Biomedical Research Institute, Samsung Medical Center, Seoul, Korea; 2 Institute for Refractory Cancer Research, Samsung Medical Center, Seoul, Korea; 3 Samsung Advanced Institute for Health Sciences and Technology (SAIHST), Sungkyunkwan University, Seoul, Korea; 4 Department of Neurosurgery, School of Medicine, Sungkyunkwan University, Seoul, Korea; 5 Department of Anatomy and Cell Biology, School of Medicine, Sungkyunkwan University, Seoul, Korea; 6 Samsung Advanced Institute of Technology, Samsung Electronics Co., Ltd., Seoul, Korea; 7 Medicinal Bioconvergence Research Center, College of Pharmacy, Seoul National University, Seoul, Korea; University of Florida, United States of America

## Abstract

Glioblastoma (GBM) heterogeneity in the genomic and phenotypic properties has potentiated personalized approach against specific therapeutic targets of each GBM patient. The Cancer Genome Atlas (TCGA) Research Network has been established the comprehensive genomic abnormalities of GBM, which sub-classified GBMs into 4 different molecular subtypes. The molecular subtypes could be utilized to develop personalized treatment strategy for each subtype. We applied a classifying method, NTP (Nearest Template Prediction) method to determine molecular subtype of each GBM patient and corresponding orthotopic xenograft animal model. The models were derived from GBM cells dissociated from patient's surgical sample. Specific drug candidates for each subtype were selected using an integrated pharmacological network database (PharmDB), which link drugs with subtype specific genes. Treatment effects of the drug candidates were determined by *in vitro* limiting dilution assay using patient-derived GBM cells primarily cultured from orthotopic xenograft tumors. The consistent identification of molecular subtype by the NTP method was validated using TCGA database. When subtypes were determined by the NTP method, orthotopic xenograft animal models faithfully maintained the molecular subtypes of parental tumors. Subtype specific drugs not only showed significant inhibition effects on the *in vitro* clonogenicity of patient-derived GBM cells but also synergistically reversed temozolomide resistance of MGMT-unmethylated patient-derived GBM cells. However, inhibitory effects on the clonogenicity were not totally subtype-specific. Personalized treatment approach based on genetic characteristics of each GBM could make better treatment outcomes of GBMs, although more sophisticated classifying techniques and subtype specific drugs need to be further elucidated.

## Introduction

Glioblastoma (GBM) is the most malignant and aggressive primary brain tumor with less than 5% 5-year survival of patients [1], [2]. Aggressive standard therapy, radical surgery plus concurrent chemo-radiation treatment based on the temozolomide (TMZ), provides palliative treatment only [3]. Moreover, recent molecular-targets against GBM show minimal promise for improved prognosis and/or prediction of response to therapy [4]–[6]. Instead, accumulating evidences of GBM heterogeneity in the genomic and phenotypic properties have potentiated personalized approach against specific therapeutic targets of each GBM patient [7]–[9].

The Cancer Genome Atlas (TCGA) Research Network has been established the comprehensive catalog of genomic abnormalities of various refractory tumors [10]. Especially, a detailed view of the genomic changes in a large TCGA GBM cohort containing 206 patient samples confirmed previously reported GBM-associated mutations such genes as EGFR, PDGFR, MET, PTEN, TP53, RB1, PIK3R1, NF1, and ERBB2 [10]. More importantly, GBM was sub-classified into 4 different subtypes (proneural, neural, classical and mesenchymal) by integrating multi-dimensional data; gene expression, somatic mutations, and DNA copy number, which had differential clinical responses to chemo-radiation therapy [10].

Genomic signature-based classification and differential clinical outcome of TCGA GBMs have provoked personalized treatment of GBMs based on their genomic characteristics. In order to find out optimal drugs that target 4 different GBM subtypes-specific genes, an integrated pharmacological network database called ‘PharmDB’ was used [11]. Previously, we developed the patient-specific orthotopic GBM xenograft animal “AVATAR” models that predict and mimic patients' molecular/histopathological phenotypes and clinical treatment responses [12]. When these mouse platforms maintain the molecular subtypes of parent GBMs, the personalized treatments based on genomic characteristics could be examined translationally. In this study, we performed preclinical validation of personalized treatments for each GBM subtype with the drugs suggested by PharmDB using the patient-derived orthotopic xenograft models representing GBM subtypes.

## Materials and Methods

### Patient Sample preparation

From May, 2004 to June 2006, 105 clinically and pathologically available GBM tumor samples were obtained from 78 patients who had medical treatment in Samsung Medical Center (SMC, Seoul, Korea). Twenty seven samples were from GBM recurrence. All tissue samples were collected with written informed consent under a protocol approved by the Institutional Review Board of the Samsung Medical Center (2010-04-004, Seoul, Korea). The median age of the patients was 50.1 years (range, 28∼76). Patients were composed of 63 males and 42 females. All patients were diagnosed as GBM by specialized neuro-pathologists, according to the WHO guidelines [1].

### Primary Cell Culture of GBM cells

Parts of the surgical samples were enzymatically dissociated into single cells, following the procedures previously reported [13]. Dissociated GBM cells were cultured in neurobasal media with N2 and B27 supplements (0.5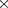 each; Invitrogen) and human recombinant bFGF and EGF (25 ng/ml each; R&D Systems) (NBE condition).

### Orthotopic Xenograft Animal Model

Animal experiments were approved by the Institutional Review Boards of the Samsung Medical Center (20131217002, Seoul, Korea) and conducted in accordance with the "National Institutes of Health Guide for the Care and Use of Laboratory Animals" (NIH publication 80–23). Acutely dissociated GBM cells were stereotactically (2 mm left and 1 mm anterior to the bregma, 2 mm deep from the dura) injected into the brains of immune deficient NOG mice within 12 hours after surgery (2.5×10^4^−1.0×10^5^ cells in 10 µl HBSS for each mice, n = 4–9 for each sample) [14]. Mice with the reduction of the total body weight (>20%) were sacrificed, and xenograft tumors were dissociated into single cells following the procedures previously reported or processed for gene expression profiling. Dissociated GBM cells were cultured in the NBE condition [13]. Some of these samples were included in the previous research (Joo et al., 2013) using same identification numbers [12].

### Gene expression profiling

mRNA expression data of 105 patient GBM samples and 25 xenograft GBM models were obtained by Affymetrix Human Gene 1.0 ST arrays. The CEL files were normalized using robust multichip average (RMA) algorithm (‘affy’ package of R 2.15.0). Probe ID annotation was processed by using GSEA-P program (downloadable from Broad Institute website). The GEO accession number for the gene expression data reported in this article is GSE58401

### GBM subtype prediction

TCGA released 840 genes which represent GBM subtypes and the mRNA expression files of 173 GBM patients [12]. The data were downloaded from https://tcga-data.nci.nih.gov/docs/publications/gbm_exp/. The 840 genes were categorized to five subtypes; 1 was assigned as proneural type, 2 as neural type, 3 as classical type, 4 as mesenchymal type, and 5 as undetermined. The Nearest Template Prediction algorithm (NTP) was used to predict the class of a given sample with statistical significance (false discovery rate, FDR<0.2) using a predefined set of markers that are specific to multiple classes [15], [16]. For in-house SMC dataset, the overlapped 788 genes among the 840 genes were used to predict the subtype.

### Drug candidate selection

To draw drug candidates for each subtype, “PharmDB” database (http://pharmdb.org) that harbors genes that can be targeted and therapeutic agents that would be associated with the possible target genes was utilized. Two therapeutic agents for each subtype were selected based on the following criteria; (1) Directly linked to at least 5 different subtype-specific genes; (2) Linked to at least 5 different subtype-specific genes via associated-proteins; (3) Linked to at least 5 different subtype-specific genes via associated-diseases; (4) Linked to at least 10% of subtype-specific genes via 2^nd^ neighboring proteins. Drugs satisfying at least one of the criteria were considered as the drug candidates, and total 8 drugs with strong evidences were selected as the final drug candidates.

### Limiting Dilution Assay

The primarily cultured GBM cells were enzymatically dissociated into single-cell suspensions, plated into 96-well plates with various seeding densities (20, 50, 100, 200, and 500 cells per well, depending on the experiments, n = 6 for each density). After seeding, the plate incubated at 37°C for 2–3 weeks. Drugs were first administered three days after the cell seeding and were added every week afterwards [Drug doses are as follows. Irinotecan Hydrochloride: 200 µM, Paclitaxel: 100 nM, Clomipramine Hydrochloride: 25 µM, Gefitinib: 100 µM, Beta-Nicotinamide Adenine Dinucleotide Hydrate: 5000 µM, Bicuculline: 2500 µM, Pravastatin Sodium Salt Hydrate: 100 µM, Resveratrol: 100 µM, Temozolomide: 1000 µM. (Every drug is diluted by 1/32 for working)]. At the time of quantification, each well was observed under a microscope for the determination of tumor sphere formation by two independent observers, blindly. When discrepancies occurred between the two, a third independent researcher decided whether the wells harbored spheres or not. For each densities of cell, ratio of wells without sphere formation was analyzed. The numbers of responded events were plotted, and tumor sphere frequency was calculated using the Extreme Limiting Dilution Analysis (http://bioinf.wehi.edu.au/software/elda/index.html). The p-value was determined by Chi-Square test compared with control group (DMSO only), and p<0.05 was considered as statistically significant.

## Results

### The ‘NTP method' predicting GBM subtype and Clinical prognosis

**Figure 1:**
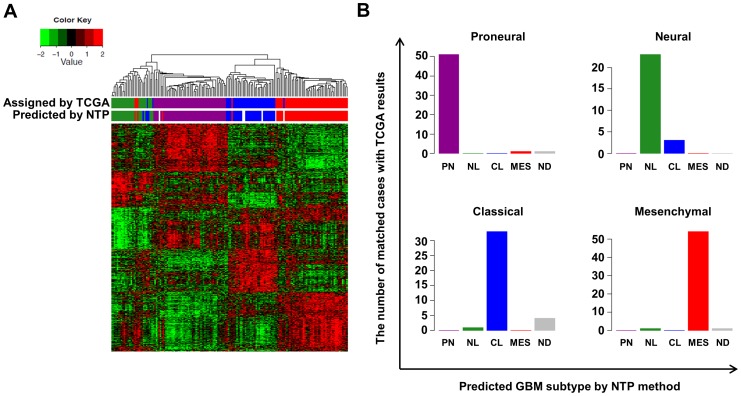
The ‘NTP method’ predicting GBM subtypes. (**A**) Hierarchical clustering using expression of 840 genes of 173 TCGA core GBM samples. The hierarchical clustering results were compared with the results of the NTP method (Predicted by NTP) and the previous reports by TCGA (Assigned by TCGA). Green, purple, blue, and red = neural (NL), proneural (PN), classical (CL), and mesenchymal (MES) subtype, respectively. (**B**) Subtype classification by the NTP methods was compared with corresponding subtypes assigned by TCGA. ND = not-determined.

**Figure 2:**
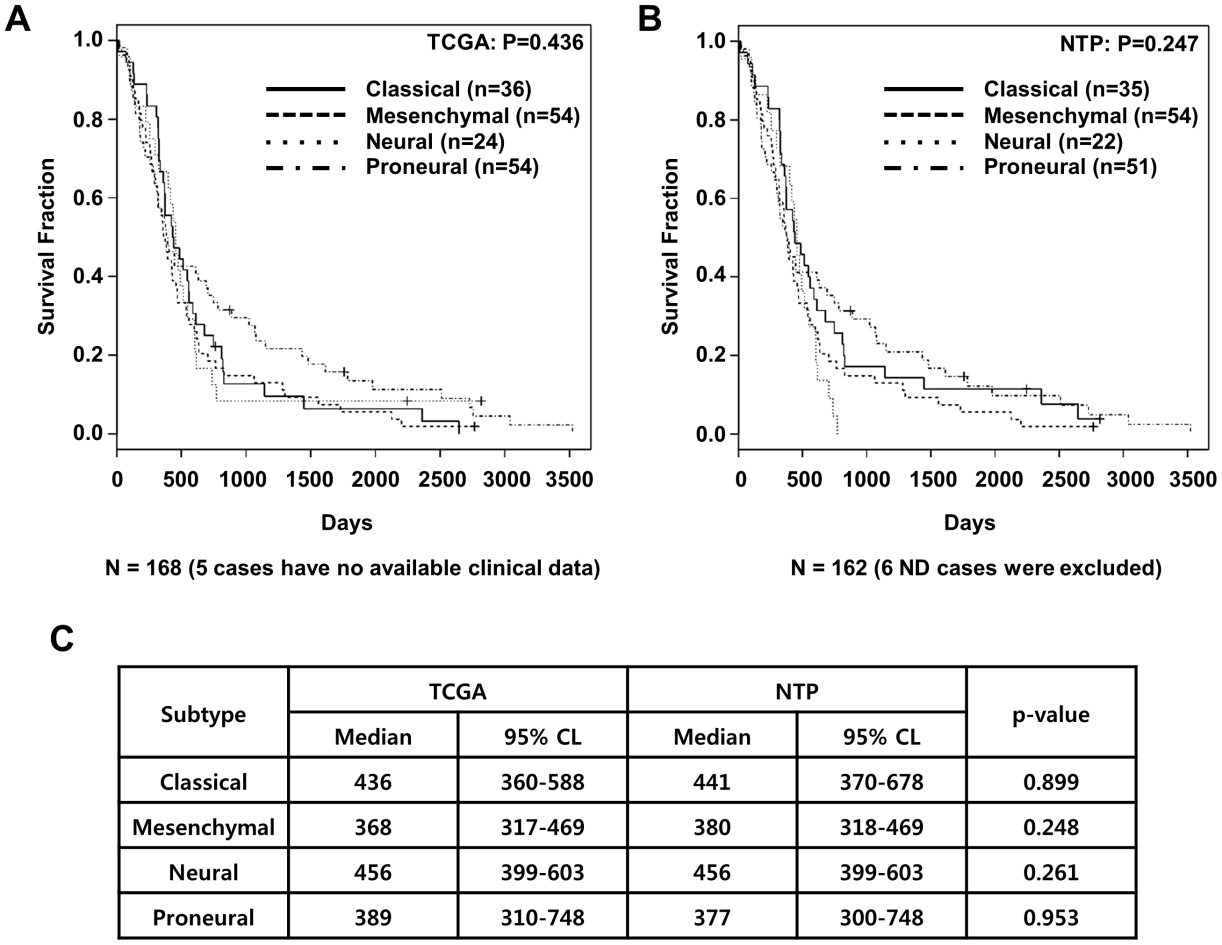
Clinical prognosis of 4 GBM molecular subtypes. 173 TCGA core GBMs' molecular subtypes were determined by TCGA group (**A**) or the NTP method (**B**). (**A–B**) Kaplan Meier curves display overall survivals of the subtypes. (**C**) Median overall survival lengths (Median) and 95% confidence limits (CL) of the subtypes determined by either TCGA group or the NTP method were compared. Log rank test was used for statistical analyses.

It is difficult to identify specific molecular subtypes of GBM xenograft tumors since mouse stromal contamination could disturb gene expression profiling that divides TCGA GBM four subtypes. To overcome those difficulties we adopted another classifying, ‘NTP method’ [15], [16]. The NTP method predicts the subtype of a given GBM or GBM xenograft sample with statistical significance (false discovery rate, FDR<0.2) using a predefined set of markers that are specific to multiple subtypes. To verify the NTP method, the 173 core GBM samples from TCGA were classified by the NTP method and compared with classification results by the original hierarchical clustering (Figure 1). 51, 23, 33, and 54 GBM cases were classified as proneural, neural, classical, and mesenchymal subtype by the NTP method, respectively. 6 cases were not classified into a specific group. Comparing with the original subtype of TCGA, total matching rate was 161/173 (93%). Using clinical data available in TCGA, clinical outcomes of GBM subtypes, classified by either hierarchical clustering or NTP method, were compared (Figure 2A and 2B). Subtype classification modified by the NTP method showed no significant alteration in clinical prognosis of each subtype (Figure 2C).

### Prognostic outcomes of 4 molecular subtypes of 105 SMC GBM patients

**Figure 3:**
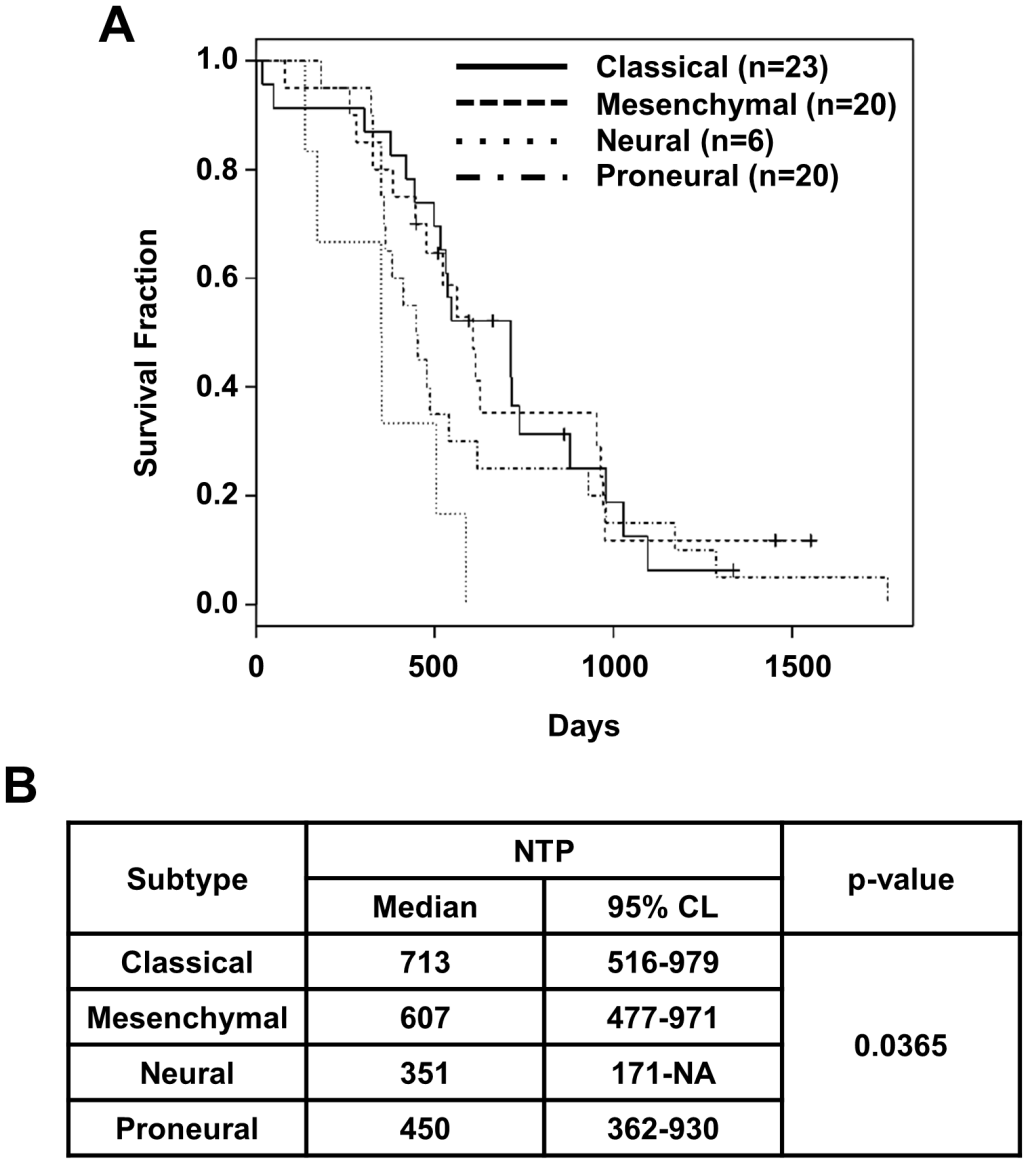
Prognostic outcomes of 4 molecular subtypes of 105 SMC GBM patients. (**A**) Kaplan Meier plot shows survivals for 4 molecular subtypes of 105 SMC GBM patients, which was predicted by the NTP method. (**B**) Median overall survival lengths (Median) and 95% confidence limits (CL) of the subtypes were summarized. Log rank test was used for statistical analyses.

The 105 GBM cases of Samsung Medical Center (SMC) GBM dataset were sub-grouped into proneural (n = 28, 26.6%), neural (n = 13, 12.4%), classical (n = 27, 25.7%), and mesenchymal subtype (n = 32, 30.5%) by the NTP method. 5 cases were not classified into a specific group. The ratio of each subtype of the SMC GBM dataset was similar with that of TCGA [the NTP method; proneural (n = 51, 25.2%), neural (n = 29, 14.4%), classical (n = 44, 21.8%), and mesenchymal (n = 49, 24.3%)]. The survival of each group was also similar with that of TCGA, although neural subtype showed a little worse clinical prognosis (Figure 3). It would be derived from insufficient neural subtype sample number in our SMC database. In the survival analysis, data from primary GBMs were utilized.

### Orthotopic xenograft “AVATAR” models recapitulate the subtypes of their parental GBMs

**Figure 4:**
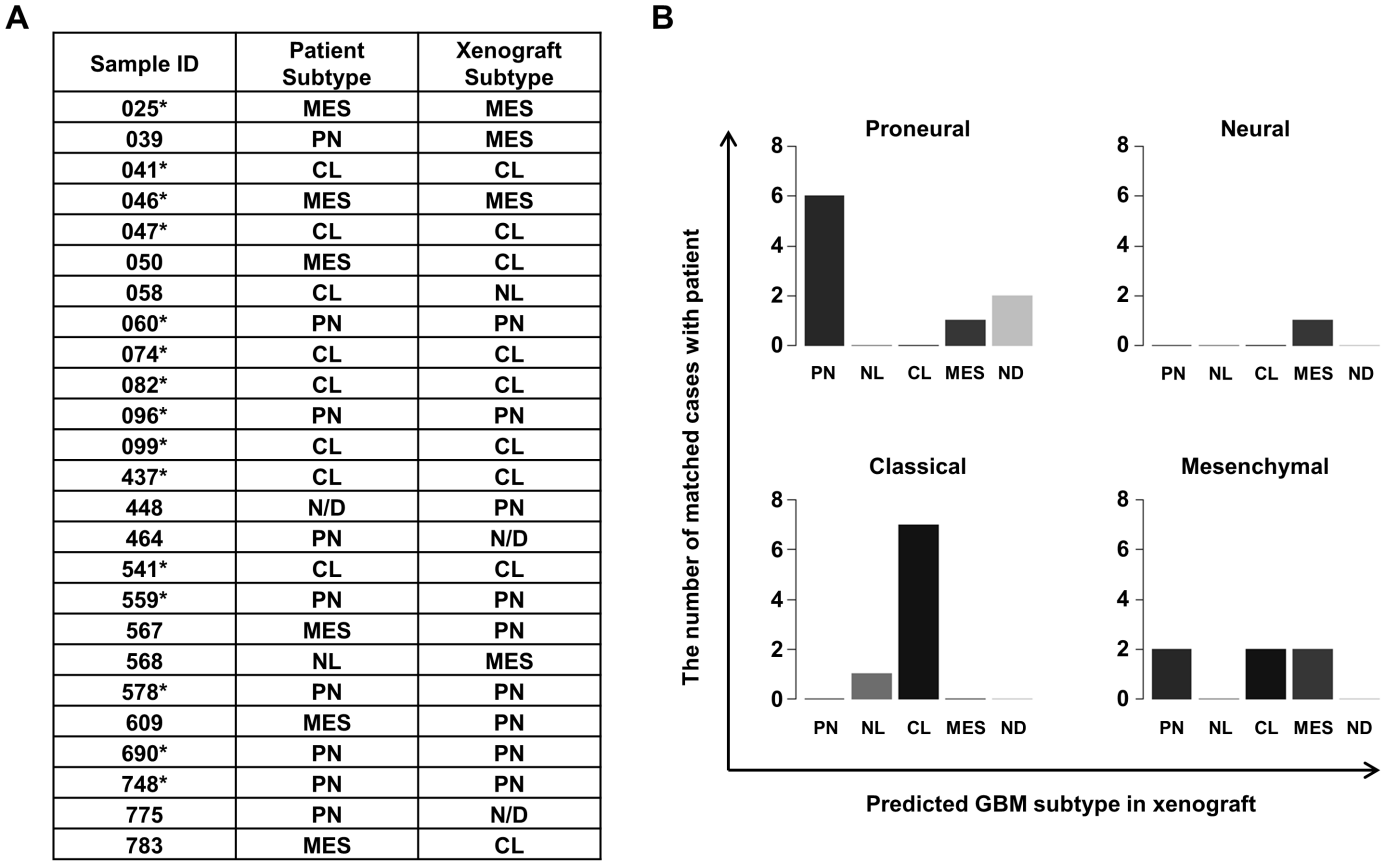
Orthotopic xenograft “AVATAR” models recapitulate the subtypes of their parental GBMs. (**A**) Predicted molecular subtypes of 25 GBM patients from the SMC GBM dataset and corresponding orthotopic xenograft “AVATAR” models were summarized. *, matched case. PN = Proneural, NL = Neural, CL = Classical, MES = Mesenchymal, ND = not-determined. (**B**) Subtype classification of 25 GBM patients by the NTP methods was compared with subtypes of corresponding orthotopic xenograft “AVATAR” models.

We have established a library of orthotopic GBM xenograft ‘AVATAR’ models using the surgical samples of SMC GBM patients [12]. The patient-specific orthotopic GBM xenograft library represents molecular and functional heterogeneity of GBMs and patient's clinical characteristics [12]. To explore whether GBM subtypes of the patients are reproduced in the xenograft models, we examined mRNA expression of 25 xenograft tumor tissues. Xenograft tumors were assigned to their accordant subtypes by the NTP method. Since subtype of 448 GBM patients was not determined by the NTP method, we could not match the subtype of xenograft tumor with that of parental tumor. As a result, 15/24 xenograft subtypes (60%) were matched with those of their parental patient samples; 6 of 9 proneural (66.7%), 0 of 1 neural (0%), 7 of 8 classical (87.5%), and 2 of 6 mesenchymal cases (33.3%), respectively (Figure 4). The high matching rates were shown in the proneural and classical subtype. However, relatively poor matching rates were observed in neural and mesenchymal cases. These discrepancies could be derived from mouse stromal cell contamination [19]–[21]. To confirm the hypothesis, H&E sections of the 25 GBM xenograft tumors were analyzed. Compared with the classical and proneural subtype, mesenchymal and neural subtype xenograft tumors showed increased mouse stromal cells (Figure S2).

### Network for GBM subtype-specific drug candidates

**Figure 5:**
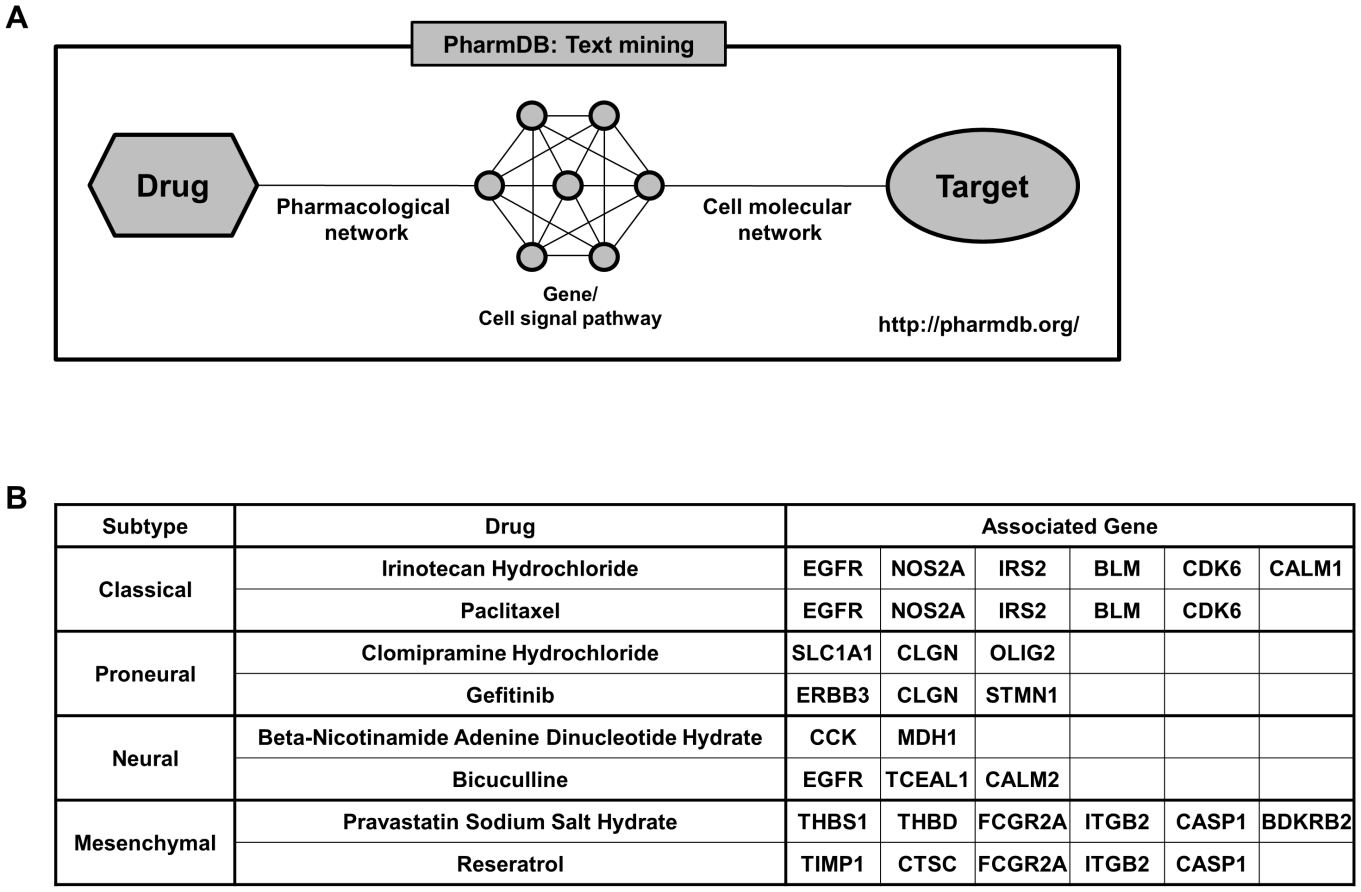
Schematization of PharmDB text mining system. (**A**) Working scheme of PharmDB that matches target genes with appropriate therapeutic agent candidates based on text mining technologies. (**B**) Subtype specific drugs and its target genes.

**Figure 6:**
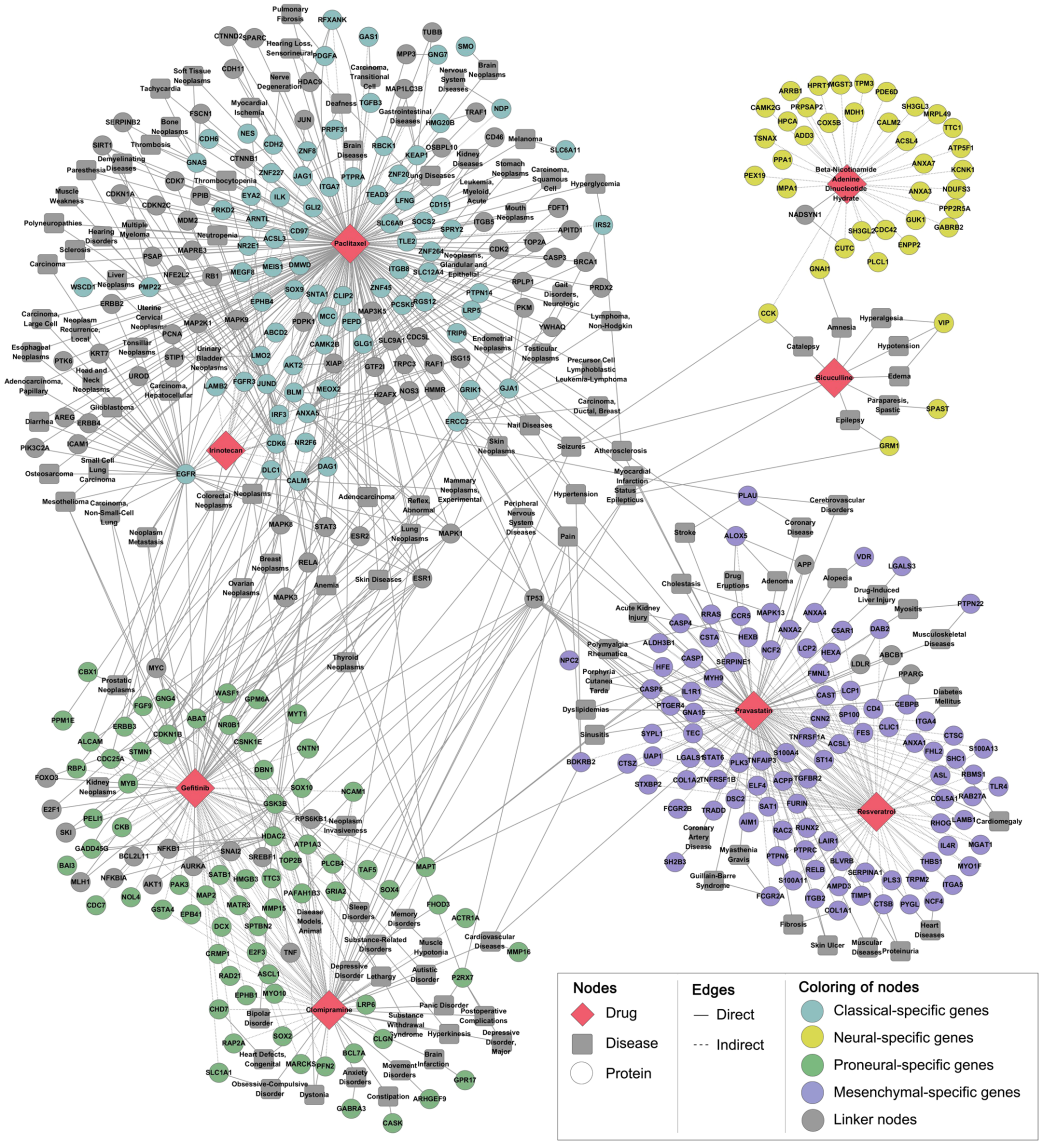
Network for GBM subtype-specific drug candidates. Eight drug candidates were directly/indirectly liked to a number of subtype-specific genes; Clomipramine: 57 proneural-specific genes; Gefitinib: 64 proneural-specific genes; Beta-Nicotinamide Adenine Dinucleotide Hydrate: 35 neural-specific genes; Bicuculline: 5 neural-specific genes; Pravastatin: 100 mesenchymal-specific genes; Resveratrol: 86 mesenchymal-specific genes; Irinotecan: 20 classical-specific genes; Paclitaxel: 79 classical-specific genes.

**Figure 7:**
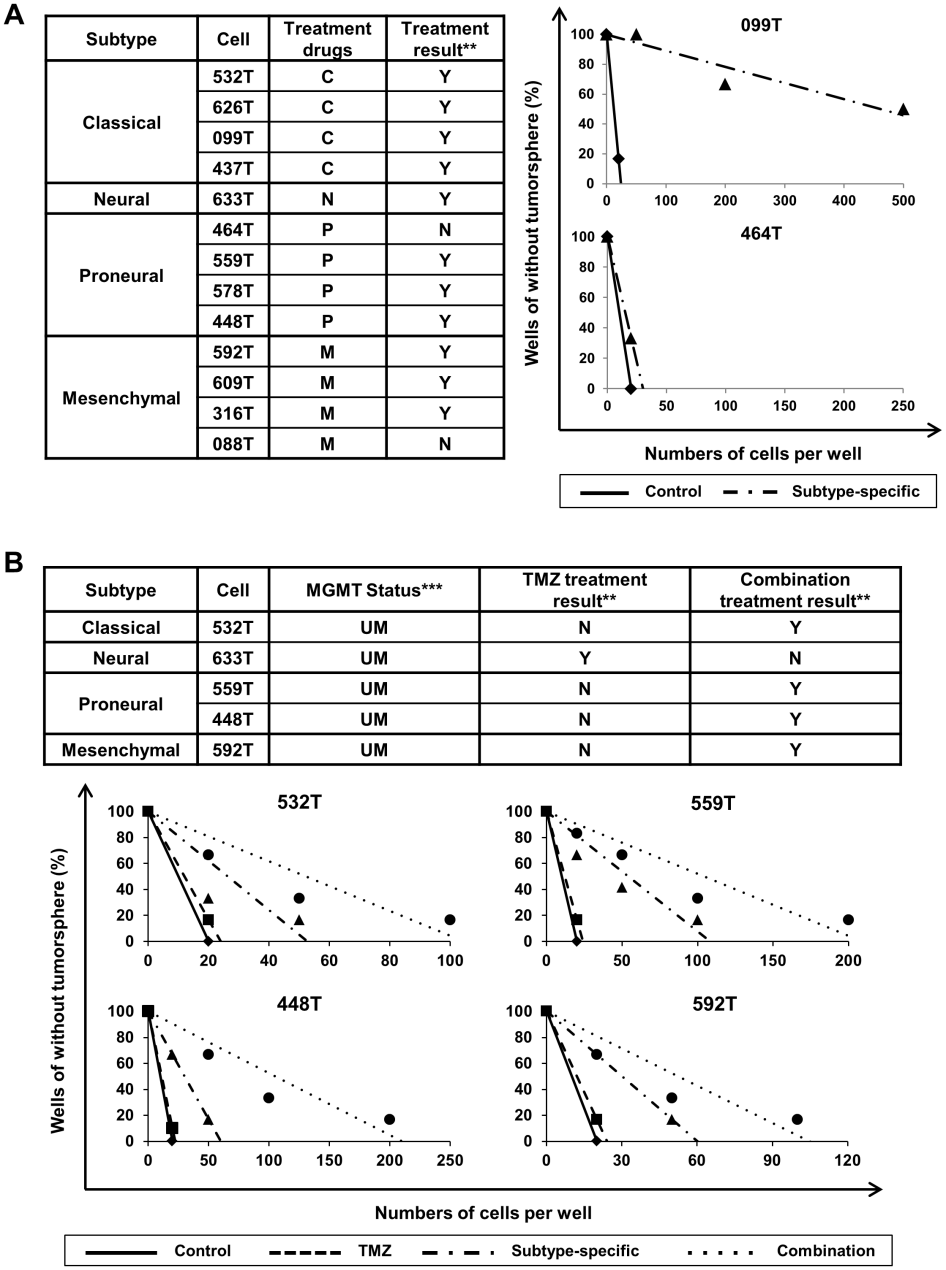
Combinational effects of molecular subtype specific drugs with TMZ on patient-derived GBM cells. (**A**) The inhibition effect of molecular subtype-specific drugs on the *in vitro* clonogenicity of matched-subtype patient-derived GBM cells was determined by limiting dilution assay in 13 patient-derived GBM cells. When the ratio of GBM cells with *in vitro* sphere-forming capacity was significantly reduced in *in vitro* limiting dilution test (Chi-Square test) compared with control group (DMSO only), the treatment was considered as “effective” [Left, **, Y = Yes (p<0.05), N = No (p>0.05)]. The two representative graphs of which has a significant effect (099T) and has no effect (464T) (Right). The experiments were triplicated and one of them was illustrated. P = Proneural specific treatment, N = Neural specific treatment, C = Classical specific treatment, M = Mesenchymal specific treatment. (**B**) MGMT methylation status of the 8 patient-derived cells is represented. ***, M = Methylated, UM = Unmethylated. The effects of combinational drug treatment (molecular subtype-specific drugs+TMZ) were determined by limiting dilution assay. When the ratio of GBM cells with *in vitro* sphere-forming capacity was significantly reduced in *in vitro* limiting dilution test (Chi-Square test) compared with control group (DMSO only) or TMZ treated group, the treatment was considered as “effective” [**, Y = Yes (p<0.05), N = No (p>0.05)]. The experiments were triplicated and one of them was illustrated.

To draw possible GBM subtype-specific therapeutic agent, we utilized “PharmDB” database that harbors genes that can be targeted and therapeutic agents that would be associated with the possible target genes (Figure 5A). We inputted the subtype-specific genes into the database (http://pharmdb.org), and, as a result, selected two drugs for each subtype that were associated with subtype-specific genes (Figure 6); Irinotecan Hydrochloride and Paclitaxel for Classical subtype, Clomipramine Hydrochloride and Gefitinib for Proneural subtype, Beta-Nicotinamide Adenine Dinucleotide Hydrate and Bicuculline for Neural subtype, and Pravastatin Sodium Salt hydrate and Reseratrol for Mesenchymal subtype (Figure 5B). To confirm the specific effect of TCGA subtype-customized drugs, we utilized 13 patient-derived GBM cells of which subtypes were determined by the NTP method based on the gene expression of xenograft tissues. The 13 patient-derived GBM cells were dissociated from the corresponding orthotopic xenograft “AVATAR” tumors. The two drugs in each subtype were applied to patient-derived GBM cells and treatment efficacy was determined by *in vitro* limiting dilution assay. When the ratio of GBM cells with *in vitro* sphere-forming capacity was significantly reduced (Chi-Square test, p<0.05) compared with control group (DMSO only), the treatment was considered as “effective”. TCGA subtype specific drugs showed significant inhibition effects on the clonogenicity of patient-derived GBM cells of each subtype in 11 cases of 13 tested cases (84.6%, p<0.05, Figure 7A and S1).

### Combinational effects of molecular subtype specific drugs with TMZ on patient-derived GBM cells

TMZ has been used as a standard chemotherapeutic drug for GBM patients and its therapeutic effect is associated with the methylation status of MGMT gene [17], [18]. We identified that 5 of the 13 patient-derived cells have unmethylated MGMT and showed *in vitro* resistance to TMZ treatment (Figure 7B). In order to find out whether the subtype specific drugs could overcome the TMZ resistance, the therapeutic effects of combination treatment with TMZ were compared to only the subtype-specific drug treatment. When the ratio of GBM cells with *in vitro* sphere-forming capacity was significantly reduced in *in vitro* limiting dilution test (Chi-Square test, p<0.05) compared with control group (DMSO only), the treatment was considered as “effective”. When we treat TMZ only on MGMT unmethylated samples; there was no effect on *in vitro* clonogenicity. However there were synergistic effects in 4 of 5 MGMT unmethylated samples, when we used TCGA subtype-specific drugs and TMZ combination (p<0.05, Figure 7B). In contrast, MGMT methylated samples had no added effects (data not shown). Together, these data support that if we could identify the MGMT methylation status and TCGA subtype of the patient, we could provide more effective personalized therapeutic options to each GBM patient.

We additionally carried out limiting dilution assay with the 4 subtype specific drugs on the 13 patient-derived cells. The ratio of GBM cells with *in vitro* sphere-forming capacity was analyzed and compared with control group (DMSO only) by Chi-Square test. When the results were rearranged to compare the p-values of treatment effects of each subtype-specific drug combinations on each patient-derived GBM cells, inhibitory effects on the *in vitro* clonogenicity were not totally subtype-specific since some of the patient-derived GBM cells were sensitive not only to their subtype specific drugs but also to other subtype specific drugs as well (Table 1). This result would indicate that we are in need of searching more specific drug combination through bioinformatics techniques and validation tools.

**Table 1:** Single treatment effects of molecular subtype specific drugs on patient-derived GBM cells.

Subtype	MGMT Status*	Cell	LDA Result (p-value)			
			Drug Type**			
			Classical	Neural	Proneural	Mesenchymal
Classical	UM	532T	0.020	1.000	0.020	1.000
	M	626T	0.043	0.093	0.000	0.001
	N/A	099T	0.000	0.000	0.000	0.000
	N/A	437T	0.000	0.000	0.236	0.000
Neural	UM	633T	0.043	0.043	0.039	0.093
Proneural	M	464T	0.236	1.000	0.093	1.000
	UM	559T	0.236	1.000	0.001	1.000
	M	578T	0.236	1.000	0.020	1.000
	UM	448T	0.236	1.000	0.004	0.009
Mesenchymal	UM	592T	0.020	0.043	0.236	0.043
	N/A	609T	0.000	1.000	1.000	0.000
	N/A	316T	0.000	0.395	0.698	0.009
	N/A	088T	0.000	0.000	0.327	0.182

*M = Methylated/UM = Unmethylated/N/A = Not applicable.

**Drug Type (Proneural = Proneural specific treatment, Neural = Neural specific treatment, Classical = Classical specific treatment, Mesenchymal = Mesenchymal specific treatment). The p-value was determined by Chi-Square test that compared with control group (DMSO only). The result “0.000” means that “<0.001”.

## Discussion

In this study, we translationally tried experimental personalized treatment based on the molecular characteristics against several patient-derived GBM cells and found that the personalized treatment could show significant inhibition effects on the *in vitro* clonogenicity and reverse the resistance to TMZ chemotherapy. The experimental personalized treatment was composed of 1) determination of molecular subtype of GBM patients 2) specific drug combinations that are associated with molecular subtype-related genes, and 3) translational platforms that mimic genetic and functional phenotype of parental patient tumors.

For the determination of molecular subtypes of parental GBMs and corresponding orthotopic xenograft tumors, we have adopted and validated a multiple classification, NTP method. If we use the NTP method, we could identify consistent subtype of not only TCGA but also our institution's GBMs. In addition, we further proved that the NTP method is compatible with classifying different types of orthotopic xenograft GBM tumors derived from GBM patients' surgical samples.

Using the NTP method, we classified our GBM patient samples by four subtypes. When we compared xenograft subtypes and those of their parental patient samples, the matching rate was 60% (15/25). Although the matching rate was relatively high in the proneural (66.7%) and classical (87.5%) subtype, neural (0%) and mesenchymal (33.3%) subtype GBMs showed low matching percent in the corresponding orthotopic xenograft tumors. We expect the reason is tumor microenvironments since the neural and mesenchymal subtype has been reported that they harbor similar gene expressional characteristics with normal neural tissue and stromal tissue, respectively [19]–[21]. In the mRNA microarray experiments using surgical samples of patients and orthotopic xenograft tumors, neural and stromal cells need to be included to make influences on the results. Moreover, because the gene expression of tumor cells could be altered in the different tumor environment, the tumor subtype could also be changed [19]–[21].

We identified molecular subtype specific drugs using a web database. Using the subtype specific drugs, we performed *in vitro* limiting dilution assay on patient-derived GBM cells that were primarily cultured from orthotopic GBM xenograft “AVATAR” animal models. The subtype specific drug showed significant inhibitory effects on the *in vitro* clonogenicity of patient-derived GBM cells. In the case of treating TCGA-subtype specific drugs combined with TMZ on MGMT-unmethylated patient-derived GBM cells provided a synergistic effect inhibiting the clonogenicity. These results display that combining the TCGA molecular subtypes and the other prognostic markers such as MGMT methylation status could be more powerful tool for discriminating GBM patients who could be candidates for personalized therapy.

Although EGFR mutations are most frequent in the classical subtype of GBM [10], gefitinib, an EGFR targeting agent, was unexpectedly selected for the proneural subtype by the web database analyzes in this study. Since EGFR gene alterations including mutations and amplifications are the most prevalent genetic events in GBM and found in >50% of GBM patients, proneural subtype GBMs also harbor EGFR mutations. Moreover, the database analyzed the relationships between drugs and the expression of many subtype specific genes (not EGFR specific mutations) [11]. Therefore, EGFR targeting agent could be selected for the proneural subtype that has fewer EGFR mutation than the classical subtype.

Recently, discrepancies between preclinical and clinical results of gene-based target drugs demand a reliable translational platform that can precisely recapitulate the biology of human cancers [22]–[32]. We have established a library of orthotopic GBM xenograft models using surgical samples of GBM patients. The patient-specific orthotopic GBM xenograft library represent the preclinically and clinically valuable “patient tumor's phenocopy” that represents molecular and functional heterogeneity of GBMs. According to the previous study, proneural, classical and mesenchymal subtypes exist in xenograft [12]. Moreover, in this study, we showed that the subtypes of orthotopic xenograft tumor are well-matched with those of parental GBMs, which would potentiate the translational value of orthotopic xenograft “AVATAR” models for personalized medicine.

In summary, we showed the possibility of personalized treatment based on gene expressional characteristics of GBMs for the first time. However, the subtype specific drugs were not perfectly specific for each subtype. Therefore, we need more sophisticated classifying techniques of GBM patients and more improved the subtype specific drug prediction methods. Based on those techniques, personalized treatment would make better clinical outcomes of GBM patients.

## Supporting Information

Figure S1 **The inhibition effect of molecular subtype-specific drugs on patient-derived GBM cells.** The inhibition effect of molecular subtype-specific drugs on the *in vitro* clonogenicity of matched-subtype patient-derived GBM cells was determined by limiting dilution assay in 13 patient-derived GBM cells. When the ratio of GBM cells with *in vitro* sphere-forming capacity was significantly reduced in *in vitro* limiting dilution test (Chi-Square test) compared with control group (DMSO only), the treatment results were represented by graphs.
(TIF)

Figure S2 **The tumor status which is derived from xenograft model in each 4 subtype.** By NTP method, 25 patient-derived xenograft tumor samples were determined each TCGA subtype. Based on the results of this, representative images of H&E (Hematoxylin&Eosin) staining were selected in each subtype-specific. The scale bar (white bar) represents 100 µM.
(TIF)
