# Supplementary figures and images for: Translational Validation of Personalized Treatment Strategy Based on Genetic Characteristics of Glioblastoma
Source: PLoS One. 2014 Aug 1;9(8):e103327. doi: 10.1371/journal.pone.0103327 (PMC4118874; doi:10.1371/journal.pone.0103327)

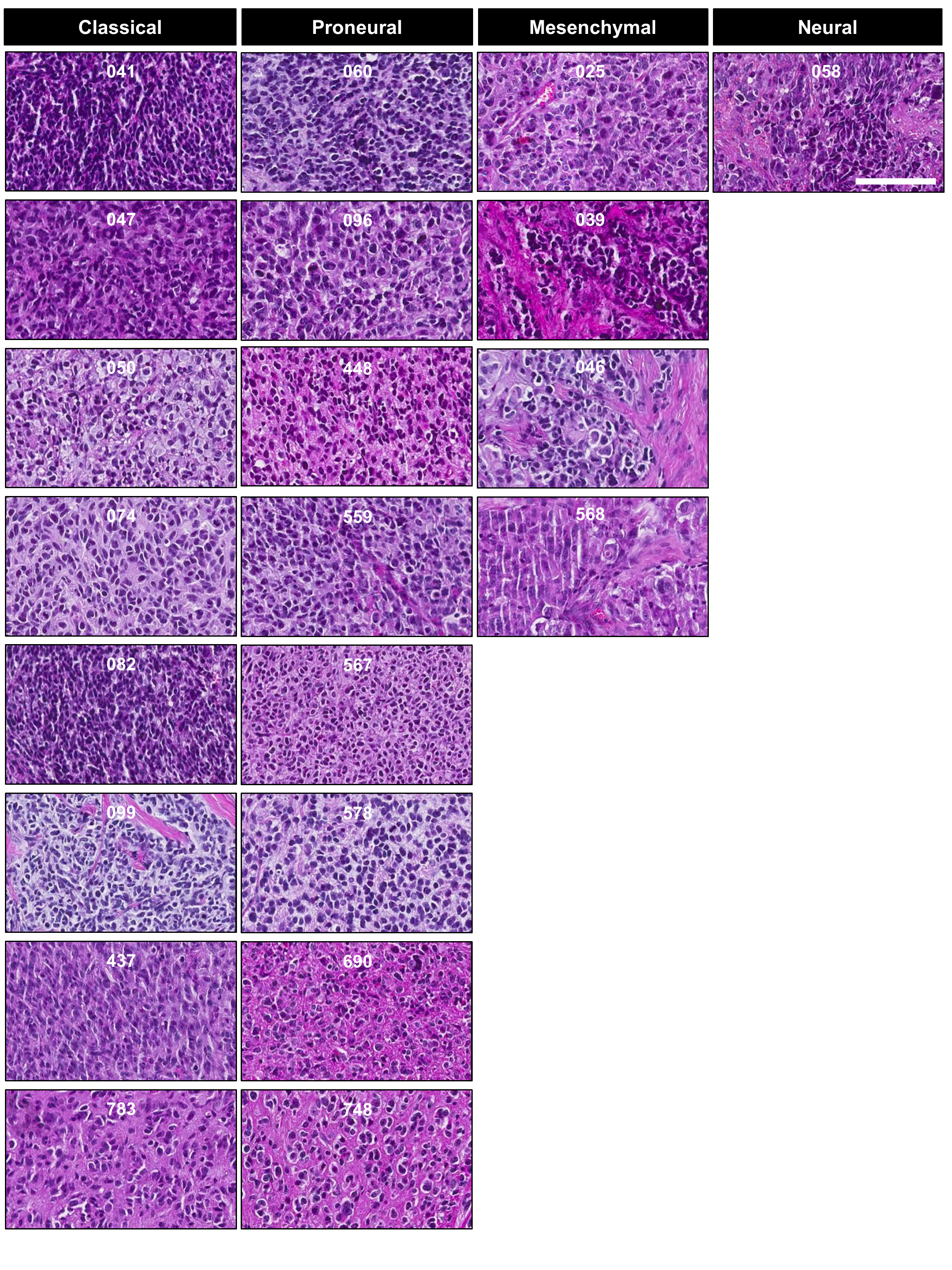

Supplement: Figure S2 — The tumor status which is derived from xenograft model in each 4 subtype. By NTP method, 25 patient-derived xenograft tumor samples were determined each TCGA subtype. Based on the results of this, representative images of H&E (Hematoxylin&Eosin) staining were selected in each subtype-specific. The scale bar (white bar) represents 100 µM. (TIF) [file pone.0103327.s002.tif]
